# Comparison of Clinical Outcomes in Patients Selected for Infra-Popliteal Bypass or Plain Balloon Angioplasty for Chronic Limb Threatening Ischemia Between 2009 and 2013

**DOI:** 10.1177/1538574420953949

**Published:** 2020-09-10

**Authors:** Matthew A. Popplewell, Huw O. B. Davies, Lewis Meecham, Gareth Bate, Andrew W. Bradbury

**Affiliations:** 1 Department of Vascular Surgery, 1724 University of Birmingham, United Kingdom

**Keywords:** chronic limb threatening ischemia, endovascular, bypass surgery, infrapopliteal, tibial

## Abstract

**Introduction:**

A published subgroup analysis of the Bypass versus Angioplasty in Severe Ischaemia of the Leg (BASIL)-1 trial suggests that, in patients with chronic limb threatening ischemia (CLTI) due to infra-popliteal (IP) disease, clinical outcomes are better following vein bypass surgery (BS) than after plain balloon angioplasty (PBA). The aim of the present study is to determine if clinical outcomes following IP revascularization in our unit are concordant with those found in BASIL-1.

**Methods:**

We analyzed prospectively gathered data pertaining to 137 consecutive CLTI patients undergoing IP PBA or BS between 2009 and 2013. We compared 30-day morbidity and mortality, days in hospital (index admission and out to 12-months), amputation free survival (AFS), overall survival (OS), limb salvage (LS), and freedom from arterial re-intervention (FFR). Patient outcomes were censored on 1 February 2017, providing a minimum 3 years follow-up.

**Results:**

Patients undergoing BS (73/137, 47%) tended to be younger, have less comorbidity, and were more likely to be on best medical therapy (BMT). BS patients spent more days in hospital during the index admission (median 9 vs 5, p = .003), but not out to 12 months (median 15 vs 13, NS). BS patients suffered more 30-day morbidity (36% vs 10%, p < .001), mainly due to infective complications, but not mortality (3.1% vs 6.8%, NS). AFS (p = .001) and OS (p < .001), but not LS or FFR, were better after BS.

**Conclusions:**

CLTI patients selected for revascularization by means of IP BS had better long-term outcomes in terms of AFS and OS, but not FFR or LS. Although we await the results of the BASIL-2 trial, current data support the BASIL-1 sub-group analysis which suggests that patients requiring revascularization for IP disease should have BS where possible and that PBA should usually be reserved for patients who are not suitable for BS.

## Introduction

Chronic limb threatening ischemia (CLTI) is a growing global health and social care problem due to widespread tobacco use and the increasing worldwide prevalence of diabetes.^1,2^ The condition remains associated with very poor outcomes in terms of major lower limb amputation (MLLA) and premature cardiovascular and all-cause mortality. The evidence base underpinning the management of CLTI is limited, especially with regard to revascularization strategies, and particularly with regard to the infra-popliteal (IP) segment. The UK National Institute of Health Research (NIHR) Health Technology Assessment (HTA)-funded Bypass versus Angioplasty in Severe Ischaemia of the Leg (BASIL)-1 remains the only published randomized controlled trial (RCT) to have compared infra-inguinal bypass surgery (BS) with plain balloon angioplasty (PBA) for CLTI.^3^

In 2017, we published a BASIL-1 IP sub-group analysis that demonstrated a trend toward improved amputation free survival (AFS) and overall survival (OS) in those undergoing BS, and showed that BS was associated with highly significantly better quality of revascularization than PBA in terms of relief of ischemic rest pain.^4^ However, the procedures were performed between 1999 and 2003 leading to suggestions that BASIL-1 outcomes are no longer relevant to current endovascular practice.

To further address the issue of preferred IP revascularization strategies for CLTI, the UK NIHR HTA funded the BASIL-2 trial which compares IP vein BS with best endovascular treatment (BET) in patients presenting with CLTI who require IP revascularization.^5^ Although the BASIL-2 trial is likely to complete recruitment in 2020, there then follows a minimum of 2 years of follow-up, such that the final results are unlikely to be published until early 2023. In the meantime, therefore, there remains a large “gray area of clinical equipoise” regarding IP revascularization strategies for CLTI.

The aim of the present study was to determine if important clinical outcomes following IP revascularization in our unit (2009-2013) are concordant with those found in BASIL-1 (2009-2013) and, further, to encourage participation in BASIL-2.

## Methods

This was a retrospective analysis of prospectively gathered hospital data pertaining to 137 consecutive CLTI patients selected for either IP PBA or BS in our unit between 1 June 2009 and 30 July 2013. Prior to revascularization, all patients were discussed in a vascular multi-disciplinary meeting. Intervention was planned based on the availability of venous conduit, patient fitness, patient choice and the technical options following detailed imaging. We compared 30-day morbidity and mortality, length of hospital stay (for both index admission and out to 12-months), amputation free survival (AFS), overall survival (OS), limb salvage (LS), and freedom from arterial re-intervention (FFR). In addition to this, we compared AFS and OS in those that survived the primary hospital episode with the index limb intact.

As the data analyzed were collected as part of normal routine clinical practice, ethical approval was not sought in accordance with advice from the UK National Research Ethics Service (http://www.hra-decisiontools.org.uk/ethics/). Patient outcomes were censored on 1 February 2017 so providing a minimum of 3 years follow-up. Continuous data are reported using mean or median values and compared using t-test or Mann-Whitney U test, depending on the characteristics of data distribution. Categorical data are compared using Fishers Exact or Chi-squared test. Time to event data were analyzed using the Kaplan-Meier method and log-rank statistics presented as a hazard ratios (HR) and 95% confidence intervals (CI). Statistical analysis was performed using R version 3.6.1 (2019-07-05)© 2019. The R Foundation for Statistical Computing. Graphical illustrations presented were prepared using GraphPad Prism 8 software ver. 8.3.0, GraphPad Software, LLC©.

## Results

Patients undergoing BS (64/137, 47%) tended to be younger with less co-morbidity and were also more likely to be on best medical therapy (BMT) (Table 1). Patients undergoing PBA had a higher incidence of previous myocardial infarction (BS 10/64, 16% vs. PBA 29/73, 40%, p = .004) and were more likely to have diabetes (BS 27/64, 42% vs. PBA 45/73, 62%, p = .02). Although the number of patients with tissue loss in both groups was similar, the PBA patients tended to have more hindfoot involvement. A higher proportion of patients undergoing BS had undergone previous intervention in the index leg (BS 24/64, 38% vs. PBA 16/73, 22%, p = .04).

**Table 1.** Baseline Characteristics.

	BS (n = 64)	PBA (n = 73)	p value
Mean and SD age (years)	73.2 (11.2)	76.9 (10.2)	0.02
Male (%)	48 (75%)	52 (71%)	NS
**Comorbidities**			
Mean serum creatinine mmol/l (SD)	103.7 (112.5)	125.3 (123.4)	NS
Stroke / Transient Ischemic Attack (%)	13 (20%)	16 (22%)	NS
Myocardial Infarction (%)	10 (16%)	29 (40%)	0.004
Angina (%)	17 (27%)	31 (42%)	0.05
End stage renal disease (%)	2 (3%)	7 (10%)	NS
Diabetes Mellitus (%)	27 (42%)	45 (62%)	0.02
Insulin dependent Diabetes Mellitus (%)	10/27 (37%)	17/45 (38%)	NS
**Clinical Presentation**			
Tissue loss (%)	44 (69%)	56 (77%)	NS
**Site of tissue loss** (single patient may have multiple sites)			
Hallux (%)	19 (30%)	24 (33%)	NS
Other toes (%)	19 (30%)	21 (29%)	NS
Forefoot (%)	6 (9%)	11 (15%)	NS
Hindfoot (%)	1 (2%)	8 (11%)	0.03
Ankle (%)	3 (5%)	3 (4%)	NS
Above ankle (%)	10 16%)	14 (19%)	NS
**Medical therapy**			
Antiplatelet agent (%)	55 (86%)	52 (71%)	0.04
Dual antiplatelet therapy (%)	8 (13%)	19 (26%)	0.04
Antihypertensive agent (%)	51 (80%)	58 (79%)	NS
Statin therapy (%)	57 (89%)	50 (68%)	0.004
Anticoagulation (%)	13 (20%)	5 (7%)	0.02
**Previous Intervention**			
Total (%)	24 (38%)	16 (22%)	0.04
Endovascular (%)	17 (27%)	11 (15%)	NS
Surgical (%)	10 (17%)	3 (4%)	0.03

In those patients undergoing PBA, 4 also received a bare metal stent (BMS). No drug coated balloons (DCB) or drug eluting stents (DES) were used. Only 6 PBA patients had more than one crural vessel treated (Table 2). Immediate technical success (defined by the operator at the time of procedure) was 90%. In 4 cases it was not possible to cross the target lesion; 2 patients had distal embolism which resulted in loss of the target vessel; and 1 patient had a residual stenosis that was not amenable to stenting.

**Table 2.** PBA Treatment Details.

		No. (n = 73)
**Arteries treated**	Superficial Femoral	27 (37%)
	Above Knee Popliteal	25 (34%)
	Below Knee Popliteal	33 (45%)
	Tibio-peroneal Trunk	26 (36%)
	Posterior Tibial	16 (22%)
	Peroneal	18 (25%)
	Anterior Tibial	27 (37%)
	Dorsalis Pedis	1 (1%)
**Number of IP vessels treated**	TPT most distal target	18 (25%)
	1 vessel	49 (67%)
	2 vessels	6 (8%)
	3 vessels	0 (-)
**Type of disease treated**	Occlusive	41 (56%)
	Stenotic	50 (68%)
	Combination	18 (25%)
**Type of intervention**	PBA	70 (96%)
	PBA + bare metal stent	4 (5%)
**Technical Failure**	Inability to cross lesion	4/7 (57%)
	Residual stenosis	1/7 (14%)
	Embolism	2/7 (29%)
	Abandoned at patient request	0 (-)
	Total technical failure	7/73 (10%)

The majority of patients undergoing BS had great saphenous venous conduit (53/64, 83%) which was usually reversed (Table 3). Prosthetic material was used in 9 patients (3 prosthetic and 6 composite-sequential grafts). Immediate technical success was 97%; 2 procedures were abandoned as the target crural vessel was judged unsuitable for bypass following surgical exposure. In 5 patients, the bypass failed within 30 days.

**Table 3.** BS Treatment Details.

		No. (n = 64)
**Conduit**	Reversed vein (when vein used alone)	34 (53%)
	Non-reversed vein (when vein used alone)	19 (30%)
	Leg vein	46 (72%) Ipsilateral GSV 6 (9%) contralateral GSV
	Arm vein	1 (2%)
	Prosthetic	3 (5%)
	Composite Sequential	6 (9%)
	No bypass performed as distal target not patent	2 (3%)
**Proximal anastomosis**	Common femoral artery	51 (80%)
	Superficial femoral artery	5 (8%)
	Profunda femoris artery	0 (-)
	Above knee popliteal artery	4 (6%)
	Below knee popliteal artery	1 (2%)
**Distal anastomosis**	Tibioperoneal trunk	8 (12%)
	Posterior tibial artery 1/3	16 (25%)
	Posterior tibial artery 2/3	4 (6%)
	Posterior tibial artery 3/3	6 (9%)
	Peroneal artery 1/3	12 (19%)
	Peroneal artery 2/3	0 (-)
	Peroneal artery 3/3	2 (3%)
	Anterior tibial artery 1/3	9 (14%)
	Anterior tibial artery 2/3	2 (3%)
	Anterior tibial artery 3/3	1 (2%)
	Dorsalis pedis	2 (3%)
**Technical failures**	Early failure (<30 days)	5 (8%)
	Abandoned, no outflow vessel	2 (3%)
	Total technical failure	7/64 (11%)

Thirty-day mortality was non-significantly worse in patients undergoing PBA (RR 2.19, 95% CI 0.44-10.91, 5 patients, 6.8% vs. 2 patients, 3.1% BS, NS). In both cohorts the most common causes of death were pneumonia, sepsis from other causes and cardiac disease (Table 4). Those selected for BS suffered more 30-day morbidity (RR 3.75, 95% CI 1.72-8.15, p < .001), mainly as a result of surgical site infection (SSI) and other causes of sepsis (**Table 5**). BS patients spent more days in hospital during the index admission (median 9 vs 5 days, p = .003), but not out to 12 months (median 15 vs 13 days, NS).

**Table 4.** Cause of Death.

Cause	BS (n = 64)	PBA (n = 73)	p value
Pneumonia/Respiratory	4 (6%)	10 (14%)	NS
Sepsis (other causes)	2 (3%)	4 (5%)	NS
Malignancy	2 (3%)	3 (4%)	NS
Cardiac	3 (5%)	9 (12%)	NS
Acute Kidney Injury	2 (3%)	0 (-)	NS
Unknown	8 (13%)	16 (22%)	NS
Stroke	2 (3%)	4 (5%)	NS
Upper Gastrointestinal bleeding	1 (2%)	0 (-)	NS
“Old age”	0 (-)	2 (3%)	NS
Intracranial bleed	0 (-)	2 (3%)	NS
Venous thromboembolism	0 (-)	1 (1%)	NS
**Total**	**24/64 (38%)**	**51/73 (70%)**	**<.001**

**Table 5.** Peri-Procedural (Thirty-Day) Morbidity.

Cause	BS (n = 64)	PBA (n = 73)	p value
Bleeding	3 (5%) (1 CD IIIb)	1 (1%) (CD II)	NS
Sepsis (urine, chest)	7 (11%) (CD II)	0 (-%)	.004
Surgical site infection (SSI)	9 (14%) (6 CD II, 3 CD IIIb)	0 (-%)	<.001
Myocardial infarction / congestive cardiac failure	3 (5%) (2 CD II), 1CD V)	4 (5%) (1 CD II, 3CD V)	NS
Groin haematoma	3 (5%) (CD IIIb)	0 (-)	NS
Venous thromboembolism	1 (2%) (CD II)	2 (3%) (1/2 CD II) (1/2 CD V)	NS
Total	23/64 (36%)	7/73 (10%)	<.001

Abbreviations; CD, Clavien-Dindo.^16^

**Figure 1.**
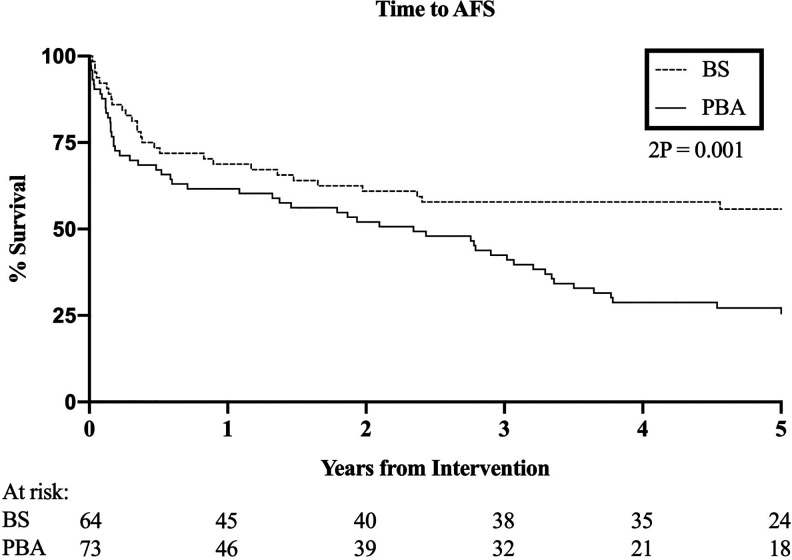
Amputation free survival. HR 0.50, 95% CI 0.33-0.77, p = .001.

AFS was significantly better in those selected for BS (HR 0.50, 95% CI 0.33-0.76, p = .001, Figure 1). AFS after BS was estimated at 69%, 58% and 56% at 1, 3 and 5 years respectively and the mean AFS after BS was 4.6 (3.8 to 5.5, 95% CI) years. AFS after PBA was estimated at 62%, 42% and 26% at the same time points and the mean AFS after PBA was 2.8 (2.2 to 3.4, 95% CI) years.

**Figure 2.**
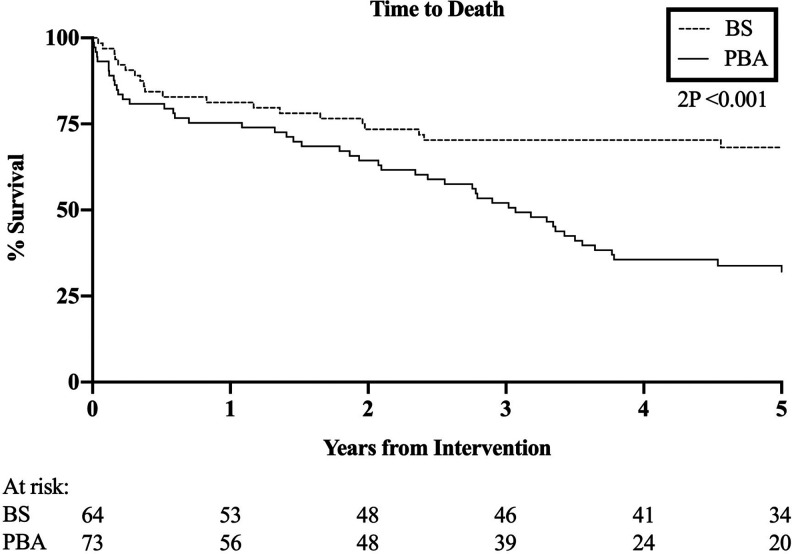
Overall survival. HR 0.42, 95% CI 0.27-0.66, p < .001.

OS was also significantly better in those selected for BS (HR 0.42, 95% CI 0.27-0.66, p < .001, Figure 2). OS following BS was estimated at 80%, 71% and 69% at 1, 3 and 5 years respectively, and the mean OS after BS was 5.6 (4.7 to 6.4, 95% CI) years. OS following PBA was estimated at 75%, 52% and 34% at the same time points, and the mean OS after PBA was 3.5 (2.9 to 4.1, 95% CI) years.

**Figure 3.**
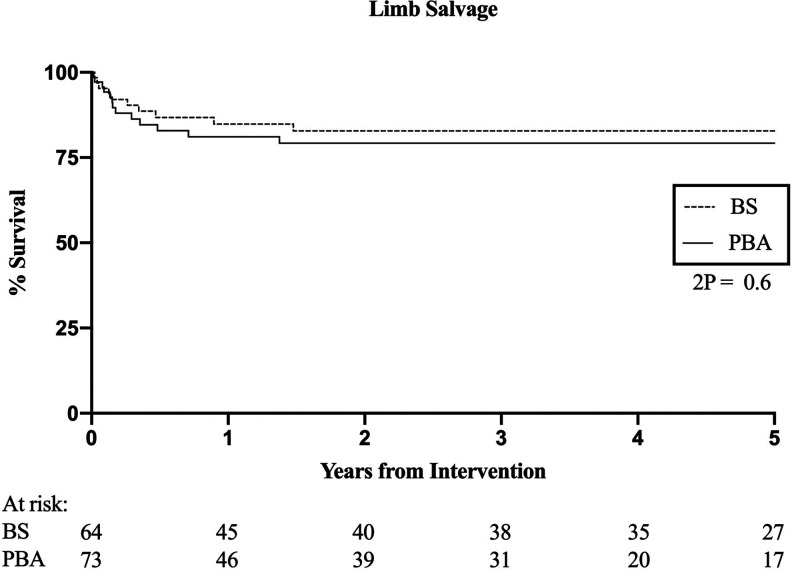
Limb salvage. HR 0.8, 95% CI 0.35-1.81, p = .6.

There was no significant difference in LS between BS and PBA (HR 0.80, 95% CI 0.35-1.81, p = .6, Figure 3). In the BS group, 6 patients had transtibial and 4 had transfemoral amputations. In comparison, 7 of those undergoing PBA required transtibial and 4 had transfemoral amputations.

**Figure 4.**
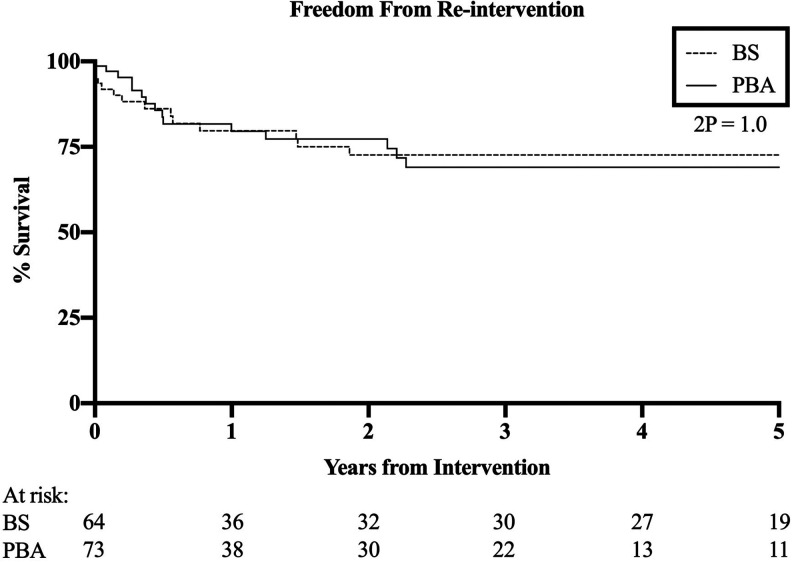
Freedom from re-intervention. HR 0.99, 95% CI 0.48-2.05, p = 1.0.

There was no significant difference in FFR between BS and PBA (HR 0.99, 95% CI 0.48-2.05, p = 1.0, **Figure 4**). Eight patients (12.5%) in the BS group required secondary bypass, and 3 (4.7%) went on to have PBA. Seven of those undergoing PBA (9.6%) had repeat PBA, and 7 patients (9.6%) went on to have BS.

Overall, 63 and 66 patients undergoing BS and PBA respectively were discharged alive from our unit without undergoing transtibial or transfemoral amputation. Of these patients, those undergoing BS had significantly better AFS (HR 0.54, 95% CI 0.35-0.84, p = .006) and OS (HR 0.46, 95% CI 0.29-0.74, p = .002) than those undergoing PBA.

## Discussion

The main finding of the present study is that important clinical outcomes following IP BS and PBA in a non-randomized contemporary series of patients treated in our unit between 2009 and 2013 are very similar, both in absolute and relative terms, to those reported in patients recruited to the multi-centre BASIL-1 RCT between 1999 and 2003. In our series, patients undergoing BS did significantly better in terms of AFS and OS than those receiving PBA. BASIL-1 reported similar outcomes but to a smaller degree of significance than we present here.

Patients in this contemporary series (CS) selected for BS were similar in terms of age and co-morbidity (previous cerebrovascular disease, myocardial infarction, end stage renal disease [ESRD] and diabetes) to those patients in the BASIL-1 IP subgroup (B1). However, those undergoing PBA tended to have a higher burden of such co-morbidity.

One may argue that the number of IP revascularizations in a 50-month period is low, given the size of our vascular unit. In the last decade we have seen a shift in the presentation of disease. A prospective cohort study performed in our unit between 2014 to 2018 to showed, disappointingly, that around 40% of patients who present with CLTI with significant IP disease are either too unfit for any intervention, present with a non-salvageable limb, or have pattern of disease that is not amenable either endovascular intervention or BS. The reasons for this are probably increasing burden of diabetes, ESRD, and advancing age of the general and CLTI population.

In terms of BMT, the prescription of statins in the current BS cohort had more than doubled when compared to the B1 BS patients (B1 39% vs. CS 89%). In the B1 subgroup, 75% of patients undergoing BS were prescribed an antiplatelet agent; in the current BS cohort this was somewhat higher at 86%. Although improved rates of statin prescription were also seen in the current PBA cohort compared with the B1 PBA patients (68% vs 25%), rates were still much lower than in those undergoing BS. Only 58% of patients undergoing PBA in the B1 cohort were prescribed an antiplatelet agent. Although this had increased to 71% in our current cohort, this is still far from optimal and may have contributed to the poorer outcomes in this group. More rigorous follow up in the BS group (graft surveillance) provides an additional opportunity to reinforce the importance of BMT. Reduced outpatient contacts with the patients undergoing PBA may explain the difference in statin prescribing practice. The recorded increase in statin prescription is likely due to an increased awareness of the importance of BMT in the CLTI population. This was further reinforced in 2012 by the National Institute of Health and Care Excellence (NICE) Clinical Guideline (CG 1457) on peripheral arterial disease.^6^

Disappointingly, the majority of CLTI patients (>80%) still present with tissue loss and this has remained unchanged since B1. It is difficult to know if the extent of tissue loss was different between these groups as the Society for Vascular Surgery (SVS) Wound Ischaemia Foot Infection (WIfI)^7^ tool was not in use during either study period. However, patients in the current PBA group tended to more tissue loss in the hindfoot, which may have contributed to their overall poorer outcomes.

We have previously reported a comparison of outcomes in the patients undergoing IP PBA in this cohort with similar patients in the B1 subgroup.^8^ In that study we demonstrated that, although patients were similar at baseline, patients undergoing IP intervention had a greater burden of FP disease in patients from the B1 cohort. Although PBA technical success was also somewhat better in our current IP cohort than B1, this did not appear to translate into any longer-term clinical benefit in terms of AFS or OS.

Immediate technical success rates have improved since BASIL-1 for both BS (97% vs 86%) and PBA (90% vs. 73%). Our current technical success for patients undergoing IP PBA is comparable to results published in the literature.^9^ This may be due to improved technologies, techniques and better patient selection.

Following PBA, 7 patients (9.6%) went on to have salvage vein BS. One may argue that this group of patients would have been better served with primary vein BS. However, how this would have affected outcomes is a matter of speculation. It is hoped that the results of the BASIL-2 trial^5^ will aide decision making in this group of patients.

Patients in our current series spent less time in hospital than those a decade previously in B1. The length of index hospital admission was almost 50% lower in our recent cohort when compared to B1 (median BS 18 days vs. PBA 10 days) for both the BS and PBA groups. B1 patients also spent longer in hospital over the 12-months following intervention (a median of over 40 days).

AFS in B1 was estimated at 36% for BS and 15% for PBA out to 5 years. This was higher in our current series (BS 56% vs. PBA 25%). Trends in OS significantly improved in those undergoing BS (B1 49% vs. CS 68%) but not PBA (B1 28% vs. CS 32%) at the same time point. Although this is likely to be multifactorial, as discussed above, it may be due in part to the better use of BMT seen in our current series. In those that survived, LS (approximately 80%) and FFR (approximately 70%) were similar to that observed for both BS and PBA in B1.

Interestingly, after removing from the analysis those patients that did not survive or underwent amputation during the index hospital admission, the significant differences in favor of BS in terms of AFS and OS remained unchanged. As in BASIL-1, these differences were most pronounced after 2 years from index intervention.

Peri-procedural (thirty-day) morbidity in the current series was very similar to that reported in B1. Thus, in the B1 BS group, 36% experienced a complication within 30-days and the majority of these were SSI and other forms of sepsis.

Unlike B1, the current cohort of patients was not randomised and so there will have been selection bias regarding the choice of revascularization. However, what the data do show is that, if a patient was deemed fit enough for BS, then long-term AFS and OS were significantly better than after PBA. This supports the findings of the BASIL-1 trial overall^10^ and the BASIL-1 IP subgroup analysis.^8^ The unanswered question is how many patients who underwent PBA in the current cohort could, and perhaps should, have undergone BS instead; and, if they had, would they have enjoyed a better longer-term outcome. Only randomised controlled trials, such as BASIL-2, that are free of selection basis can address these types of questions.

Use of drug coated balloons and drug eluting stents were not considered UK standard of care during the time of the study as there was no evidence of clinical and, in particular, cost-effectiveness.^6^ The UK NICE was criticized for this stance at the time but, more recently, significant controversy has surrounded the use of such devices owing to meta-analyses^11,12^ demonstrating an increase in all-cause mortality with paclitaxel devices. Following the publication of these data, paclitaxel-based devices were excluded from BASIL-2^5^ and recruitment was paused in both the BASIL-3 trial^13^ and SWEDEPAD^14^ trials. BASIL-3 re-opened in September 2019 and is nearing the end of recruitment. However, at the time of writing, both SWEDEPAD registry trials remain closed.

The recently published Global Vascular Guidelines^1,2^ recommend an individually tailored approach to revascularization in patients with CLTI. BS is recommended for infrainguinal disease in average risk patients with a suitable venous conduit (expected peri-operative mortality of less than 5% or life expectancy >2 years). However, these recommendations are based on the current best available evidence which is categorised by the GVG writing group as “Level C” (low). Going forward, it is very important that clinicians offer patients evidence-based revascularization (EBR) that is both clinically and, very importantly, given that the greatest future CLTI burden is likely to be in low- and middle-income countries, cost effective. The results of the BASIL-2^5^, BASIL-3^13^ and BEST-CLI^15^ randomized controlled trials are eagerly awaited.

In conclusion, in CLTI patients selected for IP revascularization at our unit, PBA was associated significantly poorer AFS and OS when compared to BS. This may be explained, at least in part, by the higher prevalence of diabetes and ischemic heart disease in this group. However, patients undergoing BS had high peri-procedural morbidity and increased length of stay. Overall, clinical outcomes in the current cohort were very similar to those reported in BASIL-1, current patients undergoing BS fared somewhat better than those undergoing PBA. Current data support the BASIL-1 IP sub-group analysis and once again suggest that CLTI patients requiring revascularization for IP disease should have BS where possible; and that PBA should usually be reserved for patients who are not suitable for BS.
